# Percutaneous Coronary Intervention for Chronic Total Occlusion—Contemporary Approach and Future Directions

**DOI:** 10.3390/jcm12113762

**Published:** 2023-05-30

**Authors:** Emil Julian Dąbrowski, Michał Święczkowski, Joanna Maria Dudzik, Oliwia Grunwald, Tomasz Januszko, Paweł Muszyński, Piotr Pogorzelski, Justyna Tokarewicz, Maciej Południewski, Marcin Kożuch, Sławomir Dobrzycki

**Affiliations:** 1 Department of Invasive Cardiology, Medical University of Bialystok, 24A Sklodowskiej-Curie St., 15-276 Bialystok, Poland; mswieczkowski1@gmail.com (M.Ś.); oliwiagrunwald@icloud.com (O.G.); tjanuszko7@gmail.com (T.J.); p.muszynski94@gmail.com (P.M.); pogo.piotr@gmail.com (P.P.); justyna.tokarewicz@gmail.com (J.T.); mpoludniewski@yahoo.com (M.P.); marcinkozuch@poczta.onet.pl (M.K.); kki@umb.edu.pl (S.D.); 2 Second Department of Nephrology and Hypertension with Dialysis Unit, Medical University of Bialystok, 24A Sklodowskiej-Curie St., 15-276 Bialystok, Poland; asiadudzik.jd@gmail.com

**Keywords:** PCI, percutaneous coronary intervention, CTO, chronic total occlusion, IVUS, ADR, CART

## Abstract

In the aging society, the issue of coronary chronic total occlusion (CTO) has become a challenge for invasive cardiologists. Despite the lack of clear indications in European and American guidelines, the rates of percutaneous coronary interventions (PCI) for CTO increased over the last years. Well-conducted randomized clinical trials (RCT) and large observational studies brought significant and substantial progress in many CTO blind spots. However, the results regarding the rationale behind revascularization and the long-term benefit of CTO are inconclusive. Knowing the uncertainties regarding PCI CTO, our work sought to sum up and provide a comprehensive review of the latest evidence on percutaneous recanalization of coronary artery chronic total occlusion.

## 1. Background

In aging societies, the issue of coronary chronic total occlusion (CTO) has become a challenge for invasive cardiologists. A recent report from Swedish Coronary Angiography and Angioplasty Registry reported that CTO was present in 16% of patients with coronary artery disease (CAD). Despite the high prevalence, percutaneous recanalization of total occlusion accounted only for 5.8% of all procedures [1]. Further analyses of the Swedish registry revealed increased mortality in patients with CTO, emphasizing the importance of the problem [2]. Based on the National Cardiovascular Data Registry report, only 3.8% of all percutaneous coronary interventions (PCI) were CTO revascularizations [3]. The seriousness of the problem is exposed even more if we add to this the fact that the procedural success rate in this group was as low as 59%.

The low rate of CTO PCI attempts and low procedural success rates in large observational reports may be related not only to the technically demanding procedure but also to the lack of clear indications included in European and American guidelines. Due to technical aspects of CTO recanalization, such procedures have been underrepresented in most of the trials regarding revascularization techniques. In the SYNTAX (Synergy Between Percutaneous Coronary Intervention with TAXus and Cardiac Surgery) trial CTO was the strongest independent predictor of incomplete revascularization [4].

Recent years have brought substantial progress in most CTO blind spots. Well-designed randomized clinical trials (RCT), large observational studies, and careful meta-analyses provided evidence on indications for intervention, procedural planning, assessment of the anatomical complexity, crossing algorithms, and finally, complication management. Our work sought to summarize and provide a comprehensive review of the latest evidence and future directions of percutaneous recanalization of coronary artery chronic total occlusion.

## 2. Indications

According to the 2018 ESC guidelines, percutaneous revascularization of CTOs should be considered in groups of patients with refractory angina or with a large area of documented ischemia within the occluded vessel territory [5]. The 2021 ACC/AHA/SCAI Guideline for Coronary Artery Revascularization state that there is uncertain benefit of PCI of a CTO in terms of improving symptoms, among patients with suitable anatomy with refractory angina on optimal medical therapy (OMT) [6]. The class of ESC recommendation is IIa with B level of evidence, which is due to the small number of randomized controlled trials and based mainly on retrospective studies, often comparing groups of patients after successful vs. unsuccessful CTO PCI. In the US guidelines, in 2021, the class of recommendations was downgraded from IIa to IIb with the level of evidence BR.

The most common difficulties in conducting randomized trials comparing CTO PCI + OMT vs. OMT alone were early termination, non-CTO lesions revascularization in both arms, and significant crossover from the OMT arm to the CTO PCI arm [7,8,9]. The randomized trials conducted so far suggest that CTO PCI + OMT may benefit patients with symptomatic angina for relief and to improve exercise capacity. Still, we need more RCTs to identify the group of patients who might benefit the most and to precisely adjust treatment.

## 3. Complexity Scores and Crossing Algorithms

### 3.1. Complexity Scores

PCI for CTO remains one of the most challenging procedures in invasive cardiology due to its perceived procedural complexity. To enhance successful procedural outcomes and lower the risk of complications it is crucial to develop pre-procedural assessments. Creating a universal scale is tough since it depends not only on the anatomical complexity of the occlusions but also on the operators’ abilities and experience. To help choose the best option of management, maximize positive results of the procedure, and improve patient’s quality of life several scores have been developed.

First and the most widely used is the J-CTO (multicenter registry in Japan) score [10] which includes five variables (occlusion length ≥ 20 mm, prior PCI attempt, CTO calcification, CTO tortuosity, blunt stump) to estimate a chance of successful guide wire crossing within 30 min. The PROGRESS-CTO (Prospective Global Registry for the Study of Chronic Total Occlusion Intervention) [11] is useful for predicting the likelihood of technical success of CTO-PCI using a hybrid approach. In comparison to J-CTO, it includes four characteristics (ambiguous proximal cap, moderate or severe proximal tortuosity, circumflex CTO, and absence of interventional collateral vessels) which make it simpler to adapt and it excludes prior PCI attempts which can be unreliable based on different experience among operators. Recently Karacsonyi et al., updated the PROGRESS-CTO score [12]. They analyzed 6946 CTO PCIs from 2016 to 2022 from 36 international centers, and they created a score of risk of a technical failure which takes under consideration similar factors to PROGRESS CTO like proximal cup ambiguity, lack of interventional collaterals but also other characteristics are included like moderate/severe calcification, age ≥65 years and lack of good distal landing zone. Not only predicting technical success scores were created but also in 2022 the PROGRESS-CTO complication score was presented which estimates the risk of MACE, mortality, pericardiocentesis, and acute myocardial infarction in patients undergoing CTO PCI [13]. The aforementioned possible complications have different criteria.

CASTLE-SCORE (coronary artery bypass graft, age, stump, tortuosity, length, the extent of calcification) [14] has the greatest discriminative capacity compared to other scores with six factors included and four of them are also part of two previous general scores (stump anatomy, tortuosity, length of occlusion, and calcification). Still, it also includes two additional variables: previous coronary artery bypass grafting (CABG) and age.

RECHARGE (Registry of CrossBoss and Hybrid Procedures in France, the Netherlands, Belgium, and the United Kingdom) [15] registry developed a score that predicts technical success based on six factors: blunt stump, lesion calcification, in-lesion tortuosity ≥45°, lesion length >20 mm, a diseased distal landing zone, and previous bypass graft on the CTO. However, only 880 patients were included in this study. Few more scores were developed, such as CL-score (Clinical and lesion-related) [16], the Ellis score [17], ORA [18], and CT-RECTOR [19] which focus on similar variables like previous ones in the prediction of technical success of CTO PCI but their use is limited to their original cohorts because their discriminatory capacity was suboptimal in different populations. That shows how complicated it is to develop a universal score. A summary of complexity scores is presented in Table 1.

### 3.2. Crossing Algorithms

CTOs are quite frequently found in angiography yet they remain without revascularization due to the perception of high failure rates and technical complexity. To help provide more successful outcomes of performing CTO PCI procedures several algorithms of crossing approach have been developed. The first was created by North American operators in 2012—the Hybrid algorithm [20]. This method is based on the possibility of rapid switching strategies during the procedure to maximize the likelihood of early successful crossing. After dual injection coronary angiography, four characteristics are considered: ambiguous proximal cap, length of the occlusion, poor distal target, and presence of appropriable interventional collaterals. Depending on these features best method of approach is chosen. This method was confirmed to be safe, efficient, and repeatable [21].

A more recent option was created in 2017, by the Asia-Pacific CTO club algorithm [22]. The main proceeding is similar to the Hybrid algorithm. This method highlights the role of intravascular ultrasound (IVUS)–guided entry to overcome proximal cap ambiguity and pays attention to preprocedural angio-CT. Moreover, the CTO—length alone does not determine what strategy should be chosen. It recommends as well using the CrossBoss catheter for in-stent CTOs and the use of parallel wiring as a bail-out strategy in the antegrade approach.

In 2019 EURO-CTO [23] club developed a modified hybrid algorithm approach. It suggests antegrade techniques to resolve proximal cap ambiguity, such as balloon-assisted subintimal entry, scratch-and-go, and IVUS-guided puncture. Furthermore, it is recommended to use parallel wiring or anterior dissection and re-entry (ADR) when antegrade wiring fails. The novelty presented in this strategy is the so-called investment procedure which refers to extra plaque modification to facilitate subsequent attempts.

In the algorithm created by Japanese CTO-PCI [24] experts in 2019, the primary antegrade approach is preferred when the score in J-CTO is 0 or when there is in-stent occlusion. On the other hand, a retrograde approach should be chosen for more severe occlusions. Finally, the switch between methods should be considered after 20 min of unsuccessful guide wiring manipulation.

**Scheme 1:**
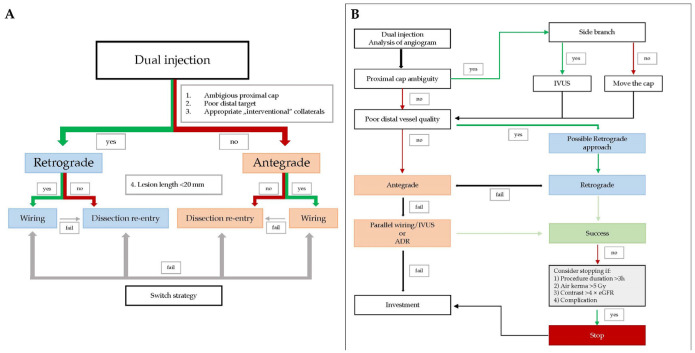
Summary of crossing algorithms: (**A**) hybrid algorithm; (**B**) global algorithm.

Recently a group of highly experienced operators from 50 countries over 4 continents indicated 7 key principles of CTO PCI [25] and developed a unified global algorithm for CTO PCI crossing [26]. It is a combination of hybrid and Asia-Pacific ways of approach, which provides more comprehensive guidelines and can simplify decisions making and enhance efficiency, safety, and repeatability in centers worldwide. The summary of selected algorithms is presented in Scheme 1.

## 4. Intravascular Imaging

Recent years brought evidence of the benefits of intravascular imaging over standard coronary angiography-guided PCI [27]. Due to the high penetration depth and no necessity for contrast media injection, IVUS is the preferred imaging method in CTO PCI. Optical coherence tomography may be useful in evaluating the need for plaque modification and stent optimization assessment. However, data on the clinical benefits, especially in terms of hard endpoints, of intravascular imaging in CTO recanalization is limited. Currently, only two RCTs investigated outcomes of intravascular-ultrasound-guided CTO angioplasty. In CTO-IVUS (Chronic Total Occlusion InterVention with drUg-eluting Stents) study IVUS-guided CTO intervention significantly reduced MACE rates at 12 months (2.6% vs. 7.1%, *p* = 0.035), however with no influence on the cardiac mortality [28]. In the angiographic endpoints-focused AIR-CTO study, Tian et al., proved that IVUS-guided CTO PCI was related to less in-stent late lumen loss and ‘in true lumen’ restenosis incidence at one-year follow-up (0.28 ± 0.48 mm vs. 0.46 ± 0.68 mm, *p* = 0.025; 3.9% vs.13.7%, *p* = 0.021, respectively) [29]. However, contrary to the results of the CTO-IVUS study, AIR-CTO failed to prove the benefit of IVUS-guided intervention in terms of adverse clinical events at one- and two-year follow-ups. Apart from the inconclusive influence on the clinical advantages of intravascular imaging, it may facilitate crossing and stent optimization during CTO PCI. In a report from the substudy of PROGRESS CTO (Prospective Global Registry for the Study of Chronic Total Occlusion Intervention), intravascular imaging was used in 38% of CTO PCI, mostly for stent sizing and stent optimization (26.3% and 38%, respectively), ante- and retrograde crossing (27.9% and 7.8%, respectively). Importantly, despite the higher complexity of lesions, intravascular imaging-guided procedures had similar technical and procedural success.

IVUS may be used for the evaluation of proximal cap ambiguity, IVUS-guided antegrade or retrograde re-entry, and evaluation of guidewire positioning (intra- or extraplaque).

### 4.1. Ambiguous Proximal Cap

According to Karatasakis, proximal cap ambiguity, defined as an inability to confidently determine the location of the proximal cap, was present in 31% of all CTO PCI and was associated with a significantly lower success rate [30]. Proximal cap ambiguity may be resolved by the performance of more angulated coronary angiography projections, the use of computed tomography, primary retrograde approach, and subintimal crossing techniques. The recent Global Chronic Total Occlusion Crossing Algorithm supports all three percutaneous strategies, suggesting that the decision should be based on angiographical images [26]. IVUS-guided puncture is the preferred approach for a CTO with a favorable side branch. After the successful puncture of a stump, IVUS provides information on the intraluminal location of the guidewire.

### 4.2. IVUS-Guided Re-Entry

IVUS guidance is effective during the ante- and retrograde subintimal techniques to re-enter the true vessel lumen after successfully crossing the occlusion. After the unsuccessful antegrade CTO crossing and distal subintimal wire position, injections should be avoided. IVUS may help direct the second wire through the plaque, confirming its position in the true lumen [31,32]. If this method fails, IVUS may be useful in finding the optimal puncture site to penetrate from extra- to intraplaque space. However, IVUS-guided antegrade re-entry is not usually a first choice, as most physicians opt TO use dedicated devices for re-entry, such as the Stingray system (Boston Scientific) [33]. Moreover, proper image interpretation may be problematic and to fit IVUS catheters and guidewire larger guiding catheters are required.

Regarding the retrograde approach, IVUS usefulness is highlighted especially in reverse controlled antegrade and retrograde tracking (CART) technique. During the passage of ante- and retrograde wires, it may confirm both wires intra- or extraplaque localization and facilitate the selection of optimal position to create a connection. Besides, IVUS provides information on true vessel diameter which is crucial for the prevention of perforation and optimal stent sizing [33].

## 5. Current Evidence

### 5.1. Randomized Clinical Trials

The potential benefits of CTO PCI remain unclear despite being practiced since the 1990s [34]. The trials investigating this matter failed to prove a positive effect on the survival of patients [7,8,9,35,36,37,38,39,40].

In the EXPLORE trial patients were randomized to CTO PCI or OMT within 7 days after primary PCI in STEMI. The study showed a trend towards a higher rate of cardiac deaths in the CTO PCI arm in 4-month follow-up [38] and in 3-year follow-up, to a significantly higher incidence of cardiac death (6.0% vs. 1.0%, *p* = 0.02) with the trend in all-cause mortality (12.9% vs. 6.2%, HR 2.07, 95% CI 0.84 to 5.14; *p* = 0.11) [41].

In the subgroup of patients with implantable cardioverter defibrillator (ICD), in EXPLORE trial, early CTO PCI after STEMI led to an increased risk of both adverse cardiac events and death [42].

The only trial that showed a decrease in major adverse coronary event rates (MACE) was the REVASC trial [35]. However, there were only three deaths reported in 12 months of follow-up and the majority of MACEs were represented by target CTO vessel revascularization incidents (14 (13.5%) vs. three (3.0%)) [35].

In CABRI trial subanalysis including 223 patients with multivessel disease and a major vessel chronically occluded (103 in the bypass group and 120 in the angioplasty group), at a median follow-up of 30 months, the incidence of death or Q-wave myocardial infarction combined were significantly lower in the bypass group (6.8% vs. 17.5%; *p* = 0.047) [43].

The achievement of complete revascularization, in both groups combined, leads to decreased death or Q-wave myocardial infarction (HR 0.26; 95% CI 0.09–0.76; *p* = 0.01) [43].

However, in the SYNTAX Extended Survival trial, subanalysis including 460 patients showed that recanalization or revascularization of total occlusions by CABG (28.0% vs. 21.4%; *p* = 0.346) or by PCI (29.9% vs. 29.4%; *p* = 0.982) did not affect the 10-year all-cause mortality as compared to patients with non-revascularized CTO, regardless of the target vessel [44].

The trials showed that the CTO PCI is most beneficial in terms of symptom relief, thus it should be considered in highly symptomatic patients, despite optimal anti-anginal therapy, without other major coronary vessels which could be a target for revascularization [8,9].

A summary of selected RCTs is presented in Table 2.

### 5.2. Observational Studies

The report from the NCDR (National Cardiovascular Data Registry) [3] discloses that the CTO PCI success rate is dependent on many variables, such as the experience of the operator and the patient’s clinical status. The procedural success was increasing progressively from 55.5% in 2009 to 61.9% in 2013. The operators’ experience was a determining component in executing a higher procedural success rate. Depending on how many procedures the operators’ carried out, the success rate (75%) was the highest for more than 10 CTO PCI procedures per year. Among those operators the median MACE rate was 1%, proving that the higher the annual volume of the operator was, the lower MACE rates were. The investigation of Habara et al. [45] strengthens this statement because higher volume centers, classified based on the operators’ experience, had higher procedural success rates—90.6% as opposed to lower volume centers—85.6%. It was mainly credited to the elevated success rates in the antegrade approach. The risk of an unsuccessful CTO PCI was exacerbated by older age, current smoking, previous MI, CABG, peripheral arterial disease, previous cardiac arrest, or right coronary artery CTO, and was alleviated by a young age and a left anterior descending artery CTO [3]. Othman et al., reported outcomes of CTO PCI among 7389 patients, who underwent PCI between January 1, 2010, and June 30, 2017 [46]. The parallel incidence rates of the MACE were elevated in the CTO PCI group in juxtaposition with non-CTO PCI patients from the registry, suggesting the importance of adequate and careful patient selection. Although in the analyzed period the rates of CTO PCI and successful recanalization increased, they remained relatively low (54.9%) as compared to high-volume CTO centers.

In the assessment of patients undergoing CTO recanalization enrolled in the European Registry of Chronic Total Occlusion between 2008 and 2015, the procedural success increased from 79.7% to an impressive 89.3% while the in-hospital mortality rate mitigated to 0.1%, despite the lesions’ complexity and emerging comorbidities [47]. The recent update of the PROGRESS-CTO registry, analyzing the outcomes of implementing hybrid algorithms reported an overall technical success rate of 86.8% with an acceptable, 3% of MACE incidence [48]. Findings of the RECHARGE Registry [49] are consistent with other analyses of dedicated CTO registries—authors reported an 86% success rate in parallel with low MACE incidence. Finally, in the OPEN-CTO Registry [50] technical success rate was 86%. The fatality index amounted to 0.9% in-hospital and 1.3% at one month. The selection of the primary strategy was determining a 60% of success rate, however, the crucial factor in elevating this ratio up to 86% was adopting the second technique. The follow-up at one month exhibited that scores, such as SAQ, the Rose Dyspnea Scale, and the PHQ-8 decreased significantly, thus early patients’ clinical status improved. The SAQ scale endpoints differentiated between successful and unsuccessful CTO PCI, which amounted to 10.8 [50]. The summary of described observational studies is presented in Table 3.

### 5.3. Meta-Analyses

RCTs and observational studies results are mostly inconsistent with each other. RCTs populations were small, follow-up was short and there was no significant difference or the results were incoherent. Researchers attempted to overcome these obstacles with patient-level and study-level meta-analyses. Some meta-analyses showed that PCI CTO resulted in reduced mortality compared to OMT. Future RCTs in bigger groups of patients are required to clarify these outcomes. The summary of selected meta-analyses is presented in Table 4.

## 6. Crossing Techniques

### 6.1. Antegrade

#### 6.1.1. Antegrade Wire Escalation

The antegrade wire escalation (AW) technique aims to reach the distal true vessel lumen by penetrating the proximal cap of the CTO by a guidewire. It is most successful with clear, straight, tapered proximal caps and short occlusions (<20 mm).

The efficiency of CTOs crossing was further increased by the development of wire with various properties regarding tip shape, presence of polymer jacket, and tip load [56,57].

After the advancement wire through the occluded artery and finding the true lumen, advancement with a microcatheter can be performed, allowing for the placement of additional equipment to continue the angioplasty procedure [56,57].

Antegrade wire escalation is successful in most cases and has less vascular compilations rate than all subintimal approaches.

**Figure 1:**
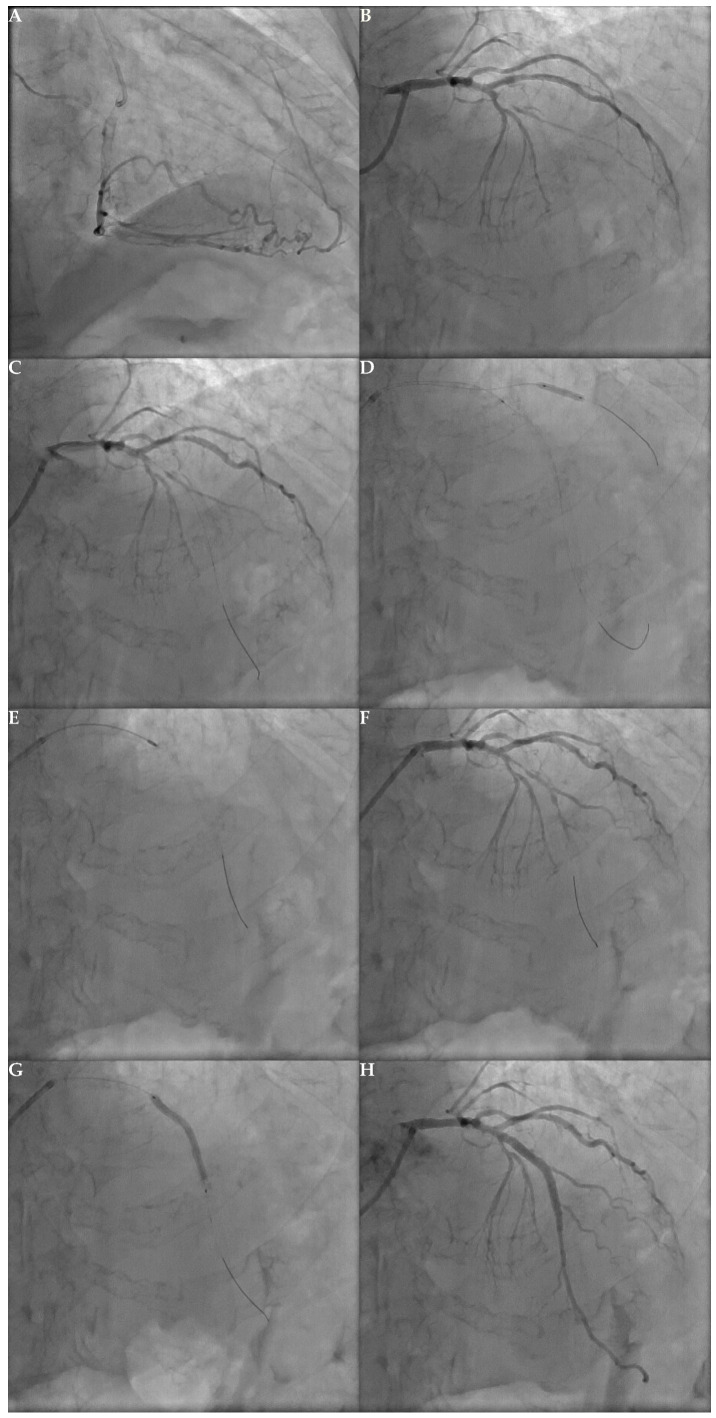
Antegrade wire escalation with use of rotational atherectomy. Panels: (**A**) Right coronary artery (RCA) injection reveals retrograde filling of left anterior descending artery (LAD). (**B**) Antegrade injections shows total occlusion of mid LAD. (**C**) Successful antegrade wiring. (**D**) Use of balloon anchoring technique. (**E**) Rotational atherectomy of severely calcified lesion. (**F**) Post-atherectomy injection. (**G**) Drug-eluting stent implantation. (**H**) Final angiographical effect.

A stepwise approach of antegrade wire escalation with the use of rotational atherectomy is presented in Figure 1.

#### 6.1.2. Antegrade Dissection and Re-Entry

Antegrade dissection and re-entry (ADR) is a technique in which the guidewire dissects through intima and later re-enters into the distal vessel’s true lumen. Primary indications are long (>20 mm) and complex occlusions. It can also be used as a secondary tool in case of incidental dissection during antegrade wiring [56,57].

ADR includes wire-based re-entry, device-based re-entry, antegrade fenestration and re-entry, and dual wire antegrade techniques [56,57].

Wire-based re-entry technique is a single wire type of ADR, firstly developed as subintimal tracking and re-entry (STAR) involving the use of a looped or ‘knuckled’ guidewire to perform dissection and advancing till entry to the distal lumen, secondly improved by advancing with a microcatheter (limited antegrade subintimal tracking and re-entry) [56,57].

Device-based re-entry techniques are based on dissecting with a dedicated catheter allowing for penetration of the distal cap with a balloon and targeted re-entry to the distal lumen [56,57].

Antegrade fenestration and re-entry techniques rely on previous preparation of the intima for dissection (balloon-assisted subintimal entry—BASE), using a balloon in a main vessel (BASE + a parallel anchor “power knuckle”), or side branch (side-balloon-assisted subintimal entry) for additional support on the guidewire during ADR [56,57].

#### 6.1.3. Parallel Wiring

Dual wire antegrade (parallel wiring) is a technique used after making a subintimal dissection. Once the soft guidewire reaches the subintimal space, it should be guided until it reaches the end of occlusion, but it should not reach the true vessel lumen. A soft microcatheter in subintimal space is also a marker and extra support for the next stiffer wire to cross the CTO. The first wire also straightens the vessel and reduces the amount of used contrast as it shows the artery’s course [56,57].

### 6.2. Retrograde

The retrograde technique outlines an attempt to cross an occlusion in a distal to the proximal manner by wiring priorly selected collateral vessels or bypass grafts to balloon and stent the aforementioned pathological lesion. Retrograde strategy in PCI may be used separately or participate in other techniques, such as controlled antegrade and retrograde subintimal tracking (CART), reverse CART, the kissing wire, and the knuckle wire technique (KWT). Determining which strategy is the best solution, is relied upon occlusion length. If it is above 20 mm, retrograde dissection, and re-entry should be implemented. If it is under 20 mm, the retrograde wire escalation will be sufficient.

It is widely established that the retrograde technique is recommended for challenging CTO lesions after a failure of antegrade crossing. Primary indications for retrograde CTO PCI are: proximal cap ambiguity, affected distal vessel, distal cap at a bifurcation, ostial occlusion, anomaly of coronary arteries, and heavy calcification.

First and foremost, it is crucial to select the most suitable collateral channel for this approach. The literature mentions that the incidence of wiring septal collaterals is about 61%, bypass grafts 13%, and epicardial collaterals 33% [58]. Bypass grafts and septal collaterals are preferred in the retrograde approach due to the lesser risk of tamponade. Fairly correspondent is a correlation between the size, the tortuosity of the collateral vessel, and the likelihood of tracking success. Best-suited prognostic factors of technical success are linked to Werner score 2 (CC2) size and lack of tortuosity of CC [59,60]. On the contrary, characteristics proven to be a vital element of unsuccessful performance are Werner score 0, tortuosity above 180°, exit angle below 90°, and collateral length [59].

The retrograde PCI technique consists of several stages, aside from them, the operator’s experience has a crucial meaning. Overall technical success was estimated at 75.2% for retrograde operators [61]. In general, the procedural success rate of the retrograde procedure can range from 80% to 84.6% [62,63,64]. However, the retrograde approach is associated with a higher risk of complications than the antegrade technique [64]. The incidence of coronary perforation in PCI CTO is about 5.5%, and 46% of it was accounted for retrograde strategy [65].

**Figure 2:**
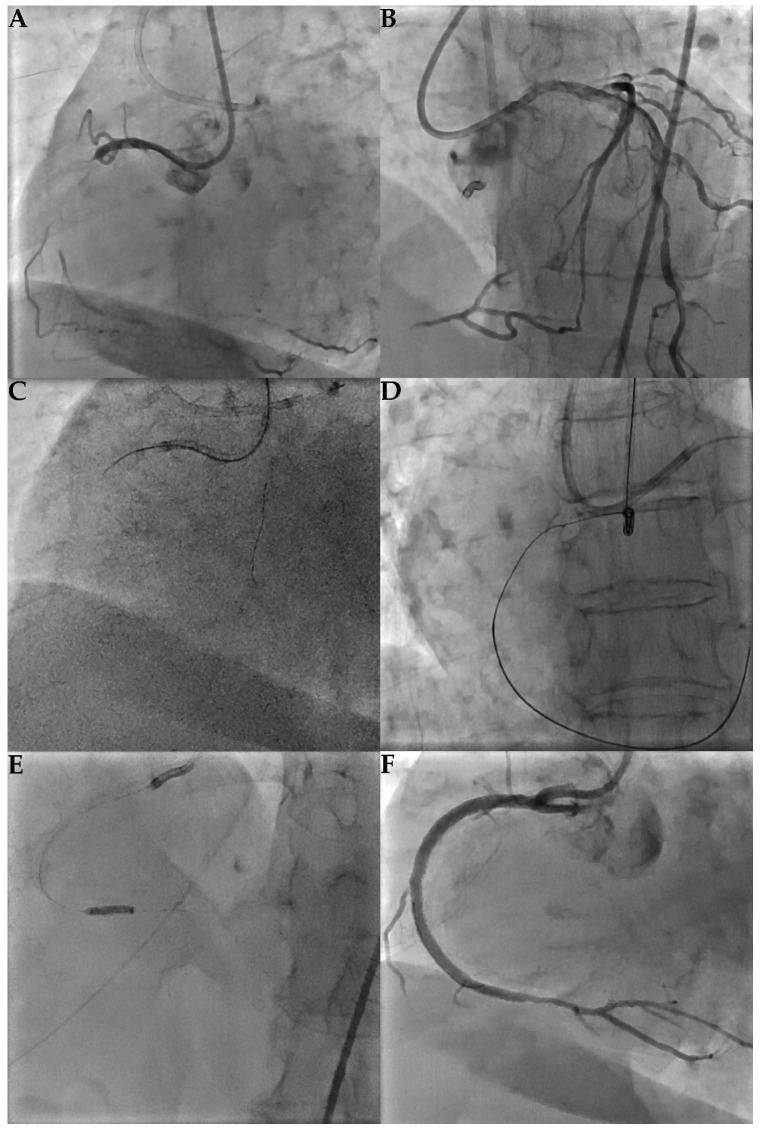
Reverse controlled antegrade and retrograde subintimal tracking. Panels: (**A**) Antegrade contrast injection reveals chronic total occlusion in the proximal right coronary artery (RCA). (**B**) Retrograde contrast injection. (**C**) Ante- and retrograde guidewires are being advanced. (**D**) Externalization of the retrograde guidewire. (**E**) Distal RCA non-compliant balloon inflation. (**F**) Final angiographic effect.

It is said that the reverse CART strategy is the most frequently used as a therapeutic retrograde measure. Reverse CART is a complexity of the bilateral approach of the CTO. To start with, the distal end of the occluded vessel should be approached through the collateral channel with the use of a retrograde workhorse guidewire, which is accompanied by a microcatheter. The selection of referred equipment must be associated with the anatomy of the vessels. To facilitate the wiring, the contrast may be administered by microcatheter and used as a guideline for the implemented wire. Insertion of the microcatheter is preceded by an inspection of the positioning of the retrograde guidewire via angiography. The following step after a positive confirmation is replacement with polymer-jacketed or spring coil wire guidewires. Simultaneously, the antegrade guidewire is being navigated proximally to the CTO, followed by the antegrade balloon inflation. After advancing the retrograde guidewire through the lesion to the proximal lumen of the target vessel, the externalization of the retrograde wire occurs. Consecutively, angioplasty should be implemented through the antegrade end. The final stage is the withdrawal of the retrograde tools. The recanalization process should be assessed, whether it was successful. Technical and procedural success outcomes for this particular technique total between 92.4% and 90.6% [66]. The stepwise approach of the reverse CART technique is visualized in Figure 2.

## 7. Complications Management

PCI CTO due to its procedural complexity has a higher complication rate (around 3% in experienced centers) than PCI performed in non-occlusive disease [48]. It is important to be aware of potential complications and to be able to recognize them in time, to minimalize morbidity [25]. Additionally, identifying patients with a higher risk of periprocedural MACE is crucial in decisions about a way of proceeding. The retrograde approach is associated with a higher risk of myocardial infarction and perforation due to more complex lesions treated with this technique. According to a study by Azzalini et al., older age, length of occlusion > 20 mm, retrograde approach, use of rotational atherectomy, and antegrade dissection and reentry techniques were associated with a greater risk of perforation [65].

CTO PCI’s complications can be categorized based on location for cardiac and noncardiac (vascular access complications, thromboembolic complications, contrast-related and radiation injury). Cardiac complications are subdivided into coronary (acute closure, perforation, and equipment loss or entrapment) and non-coronary (hypotension, myocardial infarction, tamponade, arrhythmias).

One of the most common complications is hypotension which can be caused by different factors classified as target-vessel related, donor–vessel related, and non-coronary. The first of the aforementioned group includes, among others, perforation, which must be suspected at any stage of the procedure. The incidence of perforation is higher in CTO PCI and comes to 4–9% [67]. The approach to dealing with perforation consists as below [65,68,69]:

1. Balloon occlusion proximal to the perforation side to prevent bleeding. The balloon size should be 1:1 with the target vessel and inflated at low pressure. To confirm successful execution contrast injection should be performed.
2. Intravenous fluids and pressors.
3. Pericardiocentesis in case of tamponade.
4. Urgent cardiothoracic surgery consults when percutaneous techniques fail.

Further proceeding depends on perforation localization. In large vessel perforations, stent-graft implantation is advised. In distal and collaterals vessels fat or coil embolization is used. Azzalini et al., created a registry of 1881 CTO PCIs in which perforations were observed in 5.5% with 20% of those developing tamponade [65]. A higher risk of occurring this side effect was associated with older age, occlusion length >20 mm, rotational atherectomy, antegrade dissection/re-entry, and the retrograde approach. In the American registry, the numbers were, respectively 4.1% and 14% [70]. A higher risk of perforation was observed during the retrograde approach due to its more complex and aggressive nature. Patients with perforation should be monitored in case of late tamponade development. Donor vessel-related causes of hypotension can have catastrophic outcomes. Donor vessel injury may result from deep guide engagement, thrombosis, dissection, or air embolism. When observing hypotension, firstly ensure that the guide catheter is not too deep and inspect the access site to exclude hematoma. Consider an angiogram to eliminate or confirm perforation. When present, proceed like above; otherwise, look for different causes. Donor vessel dissections usually require stent implantation, whereas donor vessel thrombosis is often successfully treated with thrombectomy. A non-coronary case of hypotension requires assessing side bleeding, and the possibility of guide interference with the aortic valve causing aortic regurgitation, vasovagal syndrome, allergic reactions, arrhythmia, or low cardiac output [69].

Other complications observed during CTO PCI are ST segment deviation, ischemia, and chest pain. Etiology can be divided into the same three groups as above: target-vessel related (deep guide engagement in the case of ipsilateral collaterals, main collateral instrumentation during the retrograde approach, no reflow or slow flow after stent deployment or post dilatation), donor-vessel target (deep guide engagement, thrombosis, dissection, air embolism), and non-coronary. It is crucial to differentiate other causes that may reflect the appearance of no-reflow such as dissection, air embolism, spasm pseudo-lesion formation, intramural hematoma, and thrombosis. For true no-reflow, several pharmacological ways of treatment have been recommended, such as adenosine, nitroprusside, nicardipine, and verapamil [69].

The third group of complications includes lost and entrapped equipment [71,72]. This is a rare situation but slightly dangerous. The way of approach is different due to the variety of equipment and multitude of mechanisms by which it could be entrapped. Retrograde devices usually need to be removed by pulling while antegrade can be removed with or without ballooning. Abandoning entrapped devices should be considered if removal is impossible or may compound the situation. Not every lost stent must be retrieved especially if it’s not located within a critical coronary location. If retrievement is needed the small balloon technique or micro snares are suggested.

CTO PCI requires higher doses of contrast which can lead to contrast-associated acute kidney injury (CA-AKI), especially for patients with chronic kidney disease (>25% in patients with severe CKD undergoing PCI) [73]. To reduce the risk of this complication, administrating intravenous fluids before and after procedures is recommended. Moreover, creating contrast-sparing protocols can reduce the risk of CA-AKI. There are several ways to reduce the amount of contrast used during the procedure such as more often using intravascular imaging, diluted contrast media, and metallic roadmapping with guidewires.

## 8. Future Directions

The main challenges lying ahead will be to develop new, efficient, and effective crossing and imaging techniques. Moreover, recommendations on managing patients with CTO still need improvements, as the latest European and American guidelines do not provide clear indications for performing PCI CTO.

### 8.1. Indications

Several studies are currently being conducted on the benefits of the procedure. The ISCHEMIA-CTO trial is designed to assess, whether percutaneous revascularization improves quality of life (QoL) and MACE in CTO patients after 6 months [74]. This study may answer questions about whether recanalization of the total occluded coronary artery improves prognosis in asymptomatic patients and/or relieve symptoms in the symptomatic group. Similarly, the NOBLE-CTO trial plans to investigate all-cause mortality and QoL in patients who underwent either PCI CTO or OMT as the initial strategy (NCT03392415) [75]. ORBITA-CTO will compare PCI CTO vs. placebo procedure and their influence on relieving symptoms of angina in patients with a previous 3-months OMT (NCT05142215) [76]. Simultaneously, studies investigating the effects of recanalization in patients with comorbidities are underway. CTO-HF trial (NCT05632653) will evaluate if PCI CTO improves survival and HF-related rehospitalizations compared to OMT, whereas the CTO-ARRHYTMIA (NCT04542460) study aims to assess the incidence of arrhythmias and the impact of PCI and OMT on them in CTO patients [77,78]. Hopefully, these aforementioned studies will provide further evidence of the benefits of PCI CTO, identify special groups in the greatest need of recanalization, and influence future guidelines.

### 8.2. Crossing Techniques

For recent decades, interventional cardiologists were waiting on a device that will break the deadlock in PCI CTO. Intravascular lithotripsy (IVL) has seen some promising results, as it was associated with 94% and 90% technical and procedural success, respectively in the PROGRESS-CTO registry [79]. For example, SoundBite Crossing System is one of the newly developed devices, which uses shockwaves to ease penetration of the proximal cap and crossing of the occlusion [80]. Moreover, intralesional delivery of collagenase (MZ-004) appears to be safe and effective in the crossing of the occlusion with a soft-tip guidewire [81]. The NovaCross, microcatheter with features like a deployable nitinol scaffold, can be used close to the proximal cap of total occlusion allowing support and centering of the guidewire through lesion penetration. In a recent clinical trial, this advanced crossing device penetrated the proximal CTO cap in 89.2% [82]. On the other hand, PlasmaWire System, a radio frequency (RF) wire system, utilizes plasma-mediated ablation to ease the crossing of the CTOs. In a study by Kanno et al., seven CTO lesions were successfully subjected to plaque ablation and channel creation without any MACCE or other minor complications [83]. Furthermore, new crossing algorithms are still being developed, less invasive, and at the same time just as effective. The minimalistic hybrid approach, which tries to merge the classic techniques of the hybrid approach, tends to minimize initial dual injection, favors wrist access, and uses smaller catheters. Initial results are promising, out of 56 performed CTO PCIs in a prospective study, there was 94.6% and 91.1% technical and procedural success, respectively [84]. A similar success rate was seen in the analysis of 143 CTO PCIs [85]. There is still a need for further research, but newly developed devices have shown some promise.

### 8.3. Imaging Techniques

One of the main obstacles to completing successful recanalization is an ambiguous course of an occluded coronary artery. However, as Hong et al., demonstrated, joint pre-procedural coronary computed tomographic angiography (CCTA) and conventional angiography during the procedure resulted in a greater success rate with fewer immediate periprocedural complications, these effects were especially prominent in patients with higher J-CTO scores [86]. Using the CCTA plaque score, which includes quantitive plaque characteristics, efficiently predicted crossing and procedural success [87]. Recently Poletti and colleagues published the first report of live computed tomography (CT) guidance in CTO PCI. In their case report, 3-dimensional CT angiography was utilized live in the cath lab [88]. It revealed an angiographically invisible vessel, resulting in successful percutaneous recanalization. It suggests that this technology may be useful in visualizing occluded vessels and adequate choice of recanalization strategy. Furthermore, clinical cases describing successful CT-guided CTO recanalization inspire some more optimism [89,90]. Currently, further clinical trials are underway to confirm the beneficial impact of using coronary CTA before PCI CTO (NCT04549896) [91].

## 9. Conclusions

Recent years brought significant improvements in procedural planning, operational techniques, crossing algorithms, and percutaneous devices. Currently, CTO PCI success rates in highly specialized centers are oscillating around 90% with complication rates as low as 0.5%. However, despite the progress and rise in rates of performed percutaneous recanalization worldwide, the clinical benefit of hard endpoints over OMT has not been established. Appropriately designed and powered RCTs that investigate the clinical benefit of CTO PCI in special groups are underway and, hopefully, more definite answers regarding improved prognosis will be provided soon. As for now, invasive treatment of CTO should be considered in patients with persisting angina despite OMT to relieve symptoms and improve quality of life and physical performance.

## Figures and Tables

**Table 1:** Complexity scores summary.

	J-CTO [10]	PROGRESS CTO [11]	Updated PROGRESS CTO [12]	CASTLE-SCORE [14]	RECHARGE SCORE [15]	CL–SCORE [16]	ORA–SCORE [18]	ELLIS–SCORE [17]
Aim	Prediction of crossing within 30 min and technical success	Prediction of technical success	Prediction of technical success	Prediction of technical success	Prediction of technical success	Prediction of technical success	Prediction of technical success	Prediction of technical success
Characteristics	1. Blunt stump	1. Ambiguous proximal cap	1. Ambiguous proximal cap	1. Stump blunt or unseen	1. Blunt stump	1. Blunt stump	1. Ostial location of CTO	1. Ambiguous proximal cap
	2. Calcification	2. Moderate or severe proximal tortuosity	2. Length ≥20 mm	2. Calcification (severe)	2. Calcification	2. Severe calcification	2. Age ≥75 years	2. Moderate/severe calcification
	3. >1 Bending within 45 degree	3. Circumflex CTO	3. Moderate/severe calcification	3. Tortuosity degree (severe or unseen)	3. Tortuosity ≥45°	3. non-LAD CTO location	3. Collateral filling Rentrop grade <2	3. Poor distal target
	4. Length > 20 mm	4. Absence of interventional collateral vessels	4. Lack of interventional collaterals	4. Length ≥20 mm	4. Length ≥20 mm	4. Length ≥20 mm		4. Length ≥10 mm
	5. Prior failed PCI		5. Age ≥65 years	5. CABG history	5. CABG history	5.CABG history		5. Collaterals straight/moderate corkscrew without kinks/tight corkscrew and/or kinked
			6. Poor distal landing zone	6. Age ≥70 years	6. Diseased landing zone	6. Previous myocardial infarction		6. Operator’s experience
								7. Ostial CTO location
Derivation/validation cases	329/165	521/260	6945	14,882/5745	880 (derivation/validation ratio 2:1)	1143/514	1073 (derivation/validation ratio 2:1)	291/145
Success	88.6% guidewire crossing within 30 min	92.9% technical success		84.2%/87.8% (derivation/validation) technical success	83%/85% (derivation/validation) technical success	Overall success rate 72.7% (procedural)	91.9% (technical)	77.9% (procedural success)
Period	2006–2007	2012–2015	2016–2022	2008–2016	2014–2015	2004–2013	2005–2014	2014–2015
Centers	12 (Japan)	7 (USA)	36 centers	55 (Europe)	17 (Europe)	2 (France)	Single centered	7 (USA, Canada)

CABG—coronary artery bypass grafting, CTO—chronic total occlusion, LAD—left anterior descending artery.

**Table 2:** Summary of selected RCTs.

Trial	DECISION-CTO [7]	CULPRIT-SHOCK [39]	EXPLORE Trial [37,38,40,42]	The IMPACTOR-CTO Trial [9]	EUROCTO Trial [8]	REVASC Trial [35]
Subject	CTO-PCI versus optimal medical therapy	Culprit-lesion-only PCI versus multivessel PCI in cardiogenic shock (CULPRIT-SHOCK)	The effect of CTO-PCI versus optimal medical therapy in patients after STEMI	CTO-PCI versus optimal medical therapy in RCA CTO	CTO-PCI versus optimal medical therapy	CTO-PCI versus optimal medical therapy
Number of patients	834 (417 CTO-PCI vs. 398 no CTO-PCI)	667 (CTO in 23.5%)	302	94 with isolated RCA CTO	396	205
Year of publication	2019	2020	2016–2017–2020	2018	2018	2018
Design	Design as a noninferiority trial to compare the effect of CTO-PCI versus no CTO-PCI, with a background of medical therapy and PCI of obstructive non-CTO lesions in both groups.	Comparison of culprit-lesion-only PCI versus multivessel PCI strategy in cardiogenic shock.	Patients after STEMI were randomized to CTO-PCI within 7 days after primary PCI or to a conservative strategy (no-CTO PCI) for at least 4 months.	Patients with RCA CTO were randomized to receive CTO PCI or optimal medical therapy with at least 2 antianginal drugs.	Design as a prospective randomised, multicentre, open-label, and controlled clinical trial comparing CTO PCI + OMT versus OMT alone. All non-CTO lesions were treated before randomization.	Design as a prospective randomised comparing CTO PCI + OMT versus OMT alone. All non-CTO lesions were treated before randomization.
Primary results	There was no difference in primary endpoint: death, MI, stroke, or any revascularization (93 (22.3%) vs. 89 (22.4%), *p* = 0.86).	Culprit-lesion-only PCI in cardiogenic shock resulted in lower rates of death or renal replacement therapy at 30 days in patients with and without CTO.	The PCI of concurrent CTO had no impact on strain recovery in patients after STEMI. CTO PCI in the LAD resulted in higher LVEF after 4 months (47.2 ± 12.3% vs. 40.4 ± 11.9%; *p* = 0.02). The incidence of 4-month major adverse coronary events was similar in both groups (5.4% vs. 2.6%; *p* = 0.25). The higher improvement in regional systolic function in the CTO territory was found in the PCI CTO arm (ΔSWT 17 ± 27% vs. 11 ± 23%, *p* = 0.03).	Inducible Myocardial Ischemia Burden deceased after RCA CTO PCI.	CTO PCI resulted in a decrease of physical limitation, angina frequency, CCS score and an improvement in quality of life.	There was no significant difference in segmental wall thickening in territories of chronic total occluded coronary arteries and all other parameters measured via cMRI.
Secondary results	3 cases of serious nonfatal complications in CTO-PCI were reported. The treatment satisfaction was measured by SAQ (1 month and 3 years after) and EQ-5D, the visual analogue scale score was higher in patients after CTO-PCI.	The presence of CTO was connected with a higher rate of death at 30 days.	There was a trend towards a higher rate of cardiac deaths in the CTO PCI group (4 (2.7%) vs. 0 (0.0%), *p* = 0.056). CTO PCI strategy was connected with a higher incidence of consecutive PCI in CTO and non-CTO vessel (39 (26.4%) vs. 20 (13.0%), *p* = 0.004). Patients with ICD had a higher risk of adverse cardiac events (32% vs. 10%, *p* < 0.01) and death (18% vs. 6%, *p* = 0.02) CTO PCI. Revascularization of a CTO after STEMI significantly shortened QT dispersion at 4 months follow-up.	Six-minute walk distance and Short Form-36 Health Survey score increased in the PCI group. There was no difference in MACE-free survival between the CTO PCI and optimal treatment groups at 12 months of follow-up (94.9% vs. 100%; *p* = 0.19).	Ischaemia-driven target revascularization was higher in the OMT group (9 (6.7%) vs. 5 (2.0%), *p* = 0.04). Major cardiovascular and cerebrovascular events, all-cause death, and cardiac death, myocardial infarction incidence did not differ between groups.	Major adverse coronary event rates at 12 months were significantly lower in the CTO PCI group (16.3% vs. 5.9%; *p* = 0.02).
Additional information	Conducted in 19 centres with an annual volume of at least 500 PCIs with the presence of a CTO operator. CTO-PCI success rate was 90.6%. The trial was terminated early because of slow patient recruitment.	-	J-CTO scores 2.1–2.3. Procedural success rate was equal to 77%.	4 (8.5%) serious nonfatal complications in CTO-PCI were reported.	J-CTO scores 1.67 - 1.82. Patients were recruited at 28 European centres with high expertise for CTO-PCI. CTO-PCI success rate at the first attempt was 83.1%. Most of the CTOs were located at RCA. Serious nonfatal complications in CTO-PCI were reported in 2.9% of subjects.	10.9% of subjects suffered from type 4 MI.

cMRI—cardiac magnetic resonance imaging, CTO—chronic total occlusion, ICD—implantable cardioverter-defibrillator, LAD—left anterior descending artery, LVEF—left ventricular ejection fraction, MACE—major adverse cardiac events, MI—myocardial infarction, OMT—optimal medical therapy, PCI—percutaneous coronary intervention, RCA—right coronary artery, and STEMI—ST-segment elevation myocardial infarction.

**Table 3:** Selected observational studies summary.

Author	N	Success, %	Technique Use, %	Final Succesful Strategy Technique	MACE, %	Death, %
Brilakis et al. [3]	22,365	58.5%	N/D	N/D	1.6%	0.4%
Habara et al. [45]	3229	88.4%	Primary antegrade approach: HC: 78.4%, LC: 76.8% Primary retrograde approach: HC 21.6%, LC: 23.2%	Primary antegrade approach: HC: 64.59%, LC: 59.77% Primary retrograde approach: HC: 15.82%, LC: 16.3%	0.53%	0.21%
Othman et al. [46]	7389	53.4%	N/D	N/D	3.3%	1.4%
Konstantinidis et al. [47]	4314	89.3%	N/D	AWE 76.9% ADR 3.6% Retrograde 19.5%	0.5%	0.1%
Maeremans et al. [49]	1253	89%	ADR 23% Retrograde 34%	AWE 58% ADR 18% Retrograde 24%	2.6%	0.2%
Sapontis et al. [50]	1000	90%	N/D	AWE 40.8% ADR 24.3% RDR 24.6% RWE 10.3%	7%	0.9%

ADR—antegrade dissection and re-entry, AWE—antegrade wire escalation, HC—high volume centre, LC—low volume centre, MACE—major adverse cardiovascular events, N/D—no data, RDR—retrograde dissection and re-entry, and RWE—retrograde wire escalation.

**Table 4:** Selected meta-analyses summary.

Author	Megaly et al. [51]	Abo-Aly et al. [52]	Iannacone et al. [53]	Megaly et al. [54]	Barbarawi et al. [55]
Type of analyzed studies	4 observational	5 observational, 3 RCTs	4 RCTs, 4 PSMs	12 observational	5 RCTs
Year of publication	2020	2019	2018	2019	2019
Number of patients	8131	4579	3971	10,240, antegrade (7451) vs. retrograde (2789)	1792
Number of PCIs	2163 prior CABG	2461	2050	10,363, antegrade (7547) vs. retrograde (2816)	N/D
PCI success rate	N/A	N/D	N/D	Antegrade vs. retrograde 80.9% vs. 87.4%; OR for procedural failure 2.16, 95% CI 1.71–2.73, *p* < 0.001	86%
Primary endpoint	Selected outcomes of CTO PCI between patients with and without prior CABG—in-hospital mortality (1), coronary perforation (2), MI (3), cardiac tamponade (4), acute cerebrovascular events (5), vascular complications (6), retrograde CTO crossing attempts (7), technical success (8).	Cardiac mortality (PCI vs. OMT)	MACE - CV death, MI, re-PCI (PCI vs. OMT)	MACE incidents: in-hospital mortality (1), MI (2), need for urgent pericardiocentesis (3), contrast-induced nephropathy (4), urgent revascularization (5), cerebrovascular events (6).	MACE (PCI vs. OMT)
Results	(1) 0.8% vs. 0.3%; OR: 2.77 [95% CI: 1.43 to 5.39]; *p* < 0.003, (2) 7.3% vs. 4.9%; OR: 2.07 [95% CI: 1.49 to 2.86]; *p* < 0.001, (3) 1.4% vs. 0.5%; OR: 2.46 [95% CI: 1.46 to 4.15]; *p* < 0.001, (4) 0.1% vs. 0.8%; OR: 0.19 [95% CI: 0.04 to 0.87]; *p* < 0.03, (5) 0.3% vs. 0.3%; OR: 1.51 [95% CI: 0.49 to 4.66]; *p* < 0.47, (6) 1.7% vs. 1.2%; OR: 1.39 [95% CI: 0.84 to 2.31]; *p* < 0.2, (7) 34.7% vs. 21.9%; *p* < 0.001, (8) 80.7% vs. 86.5%; 1.66 [95% CI: 1.42 to 1.94]; *p* < 0.001,	OR = 0.62; 95% CI = 0.42–0.93; *p* = 0.02	OR 0.76, 0.43–1.33, *p* = 0.33	(1) 0.5% vs. 0.21%; OR 2.01, 95% CI 0.91–4.43; *p* = 0.08, (2) 3.07% vs. 1.27%; OR 2.37, 95% CI 1.7–3.32, *p* < 0.001, (3) 1.07% vs. 0.42%; OR 2.53, 95% CI 1.41–4.51, *p* = 0.002, (4) 3.38% vs. 1.57%; OR 2.12, 95% CI 1.47–3.08; *p* < 0.001, (5) 0.21% vs. 0.34%; OR 0.82, 95% CI 0.30–2.25, *p* = 0.70, (6) 0.44% vs. 0.19%; OR 1.95, 95% CI 0.87–4.38; *p* = 0.11.	RR 0.83; 95% CI 0.50 to 1.36; *p* = 0.45
Secondary endpoints	N/A	All-cause mortality (1), MI (2), stroke (3), repeat revascularization (4), MACE (5).	CTO PCI vs. OMT CV death (1), AMI (2), re-PCI (3).	Long-term outcomes: all-cause mortality (1), MI (2), target lesion revascularization (TLR) (3), and target vessel revascularization (TVR) (4).	All-cause mortality, cardiac death, spontaneous myocardial infarction (MI), repeat revascularization, target vessel revascularization (TVR), stent thrombosis, and left ventricle ejection fraction (LVEF) change.
Secondary results	N/A	(1) OR = 0.60; 95% CI = 0.46–0.77; *p* = 0.001, (2) OR = 1.04; 95% CI = 0.70–1.55; *p* = 0.81, (3) OR = 0.62; 95% CI = 0.28–1.37; *p* = 0.24, (4) OR = 1.01; 95% CI = 0.58–1.76; *p* = 0.95, (5) OR = 0.76; 95% CI = 0.49–1.17; *p* = 0.21.	(1) OR 0.52, 0. 33–0.81, *p* < 0.01, (2) OR 0.91, 0.66–1.26, overall *p* = 0.61, (3) OR 1.27, 0.69–2.32, *p* = 0.44.	(1) 13% vs. 8.8%; OR 1.79, 95% CI 0.84–3.81, *p* = 0.13, (2) 5.6% vs. 2.6%; OR 2.07, 95% CI: 1.10–3.88, *p* = 0.02, (3) 12.9% vs. 7.2%; OR 2.08, 95% CI: 1.33–3.25, *p* = 0.001, (4) 32.3% vs. 17.3%; OR 1.92, 95% CI: 1.49–2.46, *p* < 0.001.	There were no significant differences between both groups with regard to all-cause mortality, cardiac mortality, spontaneous MI, stent thrombosis, repeat revascularization, or LVEF change.
Conclusion	CTOs in bypassed vessels were more calcified leading to a higher risk of procedural failure. CTO crossing in prior CABG patients often required the use of the retrograde approach, which could be associated with worse outcomes. Given the higher complexity of CTO PCIs in prior CABG patients, these procedures should ideally be performed at experienced centers by seasoned CTO operators who can promptly treat complications should they arise.	PCI was associated with lower cardiac mortality compared with OMT alone. There was no difference between the 2 strategies regarding MACE, recurrent MI, repeated revascularization, or stroke.	CTO PCI seems not to have impact on MACEs, re-PCI, and ACS. A reduction in cardiovascular mortality was observed mainly driven by PSMs.	Compared with antegrade CTO PCI, the retrograde approach is attempted in more complex lesions and is associated with a higher risk for acute and long-term complications. Judicious and skillful application of the retrograde approach remains a pillar of contemporary CTO PCI.	In conclusion, in patients with CTO, PCI was not associated with significant reductions in MACE, all-cause mortality, MI, stent thrombosis, or repeat revascularization, though there was a significantly lower incidence of TVR compared with OMT.

ACS—acute coronary syndrome, CABG—coronary artery bypass grafting, CI—confidence interval, CTO—chronic total occlusion, CV—cardiovascular, LVEF—left ventricular ejection fraction, MACE—major adverse cardiac events, MI—myocardial infarction, OMT—optimal medical therapy, OR—odds ratio, PCI—percutaneous coronary intervention, PSM—propensity score matching, RCT—randomized clinical trial, RR—risk ratio, and TVR—target vessel revascularization.

## Data Availability

Not applicable.
